# A Vacuolar Membrane Ferric-Chelate Reductase, OsFRO1, Alleviates Fe Toxicity in Rice (*Oryza sativa* L.)

**DOI:** 10.3389/fpls.2019.00700

**Published:** 2019-06-04

**Authors:** Lin Li, Lingxiao Ye, Qihui Kong, Huixia Shou

**Affiliations:** ^1^State Key Laboratory of Plant Physiology and Biochemistry, College of Life Sciences, Zhejiang University, Hangzhou, China; ^2^The Zhejiang University Affiliated 15^*st*^ Middle School in Hangzhou, Hangzhou, China

**Keywords:** rice, ferric reductase oxidase, iron excess, vacuole, iron homeostasis

## Abstract

Ferric reductase oxidase (*FRO*), the enzyme that reduced ferric iron [Fe (III)] into ferrous iron [Fe (II)], is known to play important roles in Fe absorption and homeostasis in plants that utilize a strategy I mechanism to obtain iron. Rice can use both strategies I and II for Fe uptake depending on the growth conditions. FRO is encoded by two genes in rice genome. Amino acid sequence alignment shows that Os*FRO1* contains all necessary predicted motifs for a functional FRO enzyme, whereas Os*FRO2* lacks a complete transmembrane domain at the N-terminal. Transient expression of OsFRO1: GFP protein fusion revealed that OsFRO1 is localized to the vacuolar membrane in rice protoplast. Os*FRO1* is primarily expressed in leaves and transcript abundance was decreased under excess Fe conditions. Transgenic plants overexpressing Os*FRO1* were more sensitive to Fe toxicity, in contrast RNA interference lines showed more tolerance to Fe excess stress. Furthermore, RNAi lines showed decreased Fe concentrations compared to wild type plants under Fe excess condition. Together these data show that OsFRO1 is involved in reducing ferric Fe into ferrous Fe in the vacuole, and makes the vacuolar stored Fe available to the cytoplasm through Fe (II) or chelated Fe (II) transporters. Under Fe excess condition, the downregulation of *OsFRO1* in the RNAi plants reduced the amount of Fe (II) available for cytoplasm, to alleviate Fe excess toxicity. This indicates that OsFRO1 plays an important role to maintain Fe homeostasis between the cytoplasm and vacuole in rice.

## Introduction

Iron (Fe) is an essential micronutrient for most living organisms. The transition between two oxidation states, the Fe (II) and Fe (III), makes it an indispensable element for multiple biological processes for plant growth (Connorton et al., 2017). Fe participates in various metabolic pathways including photosynthesis, respiration, chlorophyll biosynthesis, and nitrogen fixation (Kobayashi and Nishizawa, 2012; Briat et al., 2015). The redox properties also allow Fe to function as the catalytic component, in the form of heme or Fe–S clusters, in a wide variety of proteins (Guerinot and Yi, 1994).

Although abundant in the Earth’s crust Fe bioavailability is strongly restricted by the insolubility of ferric oxide present in well-aerated or alkaline soils, which is not easily accessible for uptake by plants (Mori, 1999). Fe deficiency retards plant growth and reduces crop yields, but excess Fe also cause toxicity to the plants (Halliwell and Gutteridge, 1992). Plants have two distinct Fe acquisition strategies to obtain Fe from the soil (Römheld and Marschner, 1986; Connorton et al., 2017). Non-grass plants, such as the model plant *Arabidopsis thaliana*, utilize strategy I, which is also known as the reducing strategy. When exposed to Fe-limiting conditions, plants secrete protons and phenolic compounds into the rhizosphere that lower the pH and enhance Fe (III) solubility (Santi and Schmidt, 2009). Fe (III) is reduced to Fe (II) by Ferric Reduction Oxidase 2 (FRO2) localized on the plasma membrane (Robinson et al., 1999). Afterward, Fe (II) is transported across the plasma membrane into the root epidermal cells by the iron-regulated transporter 1 (IRT1) (Eide et al., 1996; Varotto et al., 2002; Vert et al., 2002). Grass plants, such as barley and maize, utilize strategy II which is also called chelating strategy to take up Fe from the soil. In strategy II plants phytosiderophores (PS) are released into the rhizosphere (Kobayashi et al., 2010). After binding Fe (III) in the soil, Fe (III)-PS complexes are taken up by specific transporters belonging to the yellow stripe (YS) or YS like (YSL) family of proteins (Curie et al., 2001; Inoue et al., 2009). Rice has functional IRT1 and IRT2 that allow it to take up Fe (II) directly under submerge conditions (Ishimaru et al., 2006). In addition, rice can secrete phenolic compounds to chelate Fe (III) and solubilize apoplasmic Fe (Bashir et al., 2011; Ishimaru et al., 2011). Thus, rice is considered to use a mixed model for Fe uptake.

Under anaerobic conditions and low pH in flooded soils Fe is present mainly as soluble Fe (II) due to the low redox potential (Becker and Asch, 2005). Fe (II) can be taken up excessively by plant roots, resulting in cellular Fe overloaded, which can be toxic for plants. Fe toxicity has become a widespread nutrient stress affecting the growth of wetland rice, especially in Asia and West Africa (Sahrawat, 2004; Audebert and Fofana, 2009). In plant cells excess amount of Fe (II) contributes to the formation of hydroxyl radicals (⋅OH) and other reactive oxygen species (ROS) through the Fenton reaction (Fenton, 1984; Becana et al., 1998). These hydroxyl radicals can react with lipids, DNA and proteins, causing irreversible damage and oxidative stress (Briat et al., 1995; Fang et al., 2001). In rice the most obvious symptoms of Fe-overloading stress are leaf bronzing (Asch et al., 2005; Becker and Asch, 2005). Moreover, excess Fe in the soil can damage the root uptake system and adversely affect the acquisition of other nutrients, such as phosphorus, zinc and magnesium, leading to reduced growth and yield loss and even death of plants (Sahrawat, 2004). Thus, the cellular Fe level must be tightly controlled to maintain Fe homeostasis in plants.

The mechanisms for Fe excess tolerance can be divided into three aspects: (1) Avoiding excess Fe accumulation – Fe deposits at the root surface as the Fe plaque and forms a physical barrier to exclude excess Fe uptake (Deng et al., 2010). Reduced Fe translocation from roots to shoots could be important to prevent oxidative stress in leaves. (2) Storing high Fe levels – Fe partitioning into different sub-cellular compartments, mainly apoplast and vacuoles, are crucial to alleviate Fe toxicity (Moore et al., 2014). Ferritins can store up to 4,000 Fe atoms and are considered essential for tolerance to excess Fe (Briat et al., 2010). (3) Scavenging of ROS by antioxidants – Plants may also utilize antioxidants such as ascorbate, glutathione, phenolics or antioxidant enzymes such as superoxide dismutase (SOD), ascorbate peroxidase (APX), and catalase (CAT) to detoxify oxidative molecules (Fang et al., 2001; Wu et al., 2017).

A significantly amount of work has been done on Fe-deficiency in plants. In contrast, the molecular aspects of Fe excess responses remain largely unknown. Several quantitative trait loci (QTL) co-localized on chromosome 1 were reported to be associated with tolerance to Fe excess stress in rice (Wu et al., 2014; Dufey et al., 2015). These results were also confirmed by genome-wide association study (GWAS) (Matthus et al., 2015). A transcriptomic study compared the expression profiling of rice seedlings after short- and long-term exposure to Fe excess (Quinet et al., 2012). It was found that more genes were up- or down-regulated after 3 days than after 3 weeks of Fe excess treatment. Transcriptomic analysis of rice in response to Fe deficiency and excess revealed that there is crosstalk between the two treatments and small open reading frames might play an important role in the response of plants to Fe deficiency and excess (Bashir et al., 2014). OsWRKY80 is the first reported transcription factor induced by Fe excess in plants (Ricachenevsky et al., 2010). However, the biological function of this gene is still under investigation.

Ferric reductase oxidase (FRO), the enzyme to reduce Fe (III) into Fe (II), is known to play important roles in Fe absorption and homeostasis in strategy I plants. There are two *FRO* genes in rice genome, but their function has not yet been characterized. In this study, the predicted amino acid sequence alignment shows that OsFRO1 contains all necessary motifs for a functional FRO enzyme. OsFRO1 is localized in the vacuolar membrane, which is different from other plant FRO proteins previously reported. Under Fe excess conditions, the transcript abundance of Os*FRO1* was decreased in abundance. Knockdown of *OsFRO1* by RNAi alleviated Fe toxicity in transgenic rice. This work indicates that OsFRO1 plays an important role to maintain Fe homeostasis in rice.

## Materials and Methods

### Plant Materials and Growth Conditions

The rice cultivar (*Oryza sativa L.* ssp. *japonica* cv. *Dongjin*) was used for physiological experiments and rice transformations. Seeds were germinated in water for 3 days. After germination uniform seedlings were transferred to nutrient solution containing 1.425 mM NH_4_NO_3_, 0.323 mM NaH_2_PO_4_, 0.513 mM K_2_SO_4_, 0.998 mM CaCl_2_, 1.643 mM MgSO_4_, 0.009 mM MnCl_2_, 0.075 mM (NH_4_)_6_Mo_7_O_24_, 0.019 mM H_3_BO_3_, 0.155 mM CuSO_4_, 0.152 mM ZnSO_4_, 125 μM Fe (II)-EDTA, with pH 5.5 (Yoshida et al., 1976). For gene expression assay, 2-week old seedlings were transferred to normal, Fe deficient or excess nutrient solutions. The solution contains 0, 0.125 mM Fe or 1 mM Fe (II)-EDTA, respectively. Plants were harvested at 7 days after Fe treatment. For phenotypic analysis, 2-week-old seedlings were grown in nutrient solution with 7 or 0.125 mM Fe (II)-EDTA for 3 days.

All the experiments were carried out in green house with a day/night temperature of 30°C/28°C and a 12 h photoperiod. The nutrient solution was changed every 3 days.

### Transient Expression of OsFRO1-GFP and OsFRO2-GFP Fusion Protein

To investigate the subcellular localization of OsFRO1 and OsFRO2, the coding sequences of *OsFRO1* and *OsFRO2* without a stop codon was amplified and cloned into pDEST/CGFP using the gateway system. Rice protoplasts were isolated from 2-week-old seedlings using cellulose R10 and macerozyme R10 as previously described (Ying et al., 2017). Two hundred micro liter of protoplast suspension was transformed with 8∼10 μg plasmid DNA using the PEG (polyethylene glycol)-mediated transformation method (Chen et al., 2011). After incubation at 25°C in the dark for 12 to 14 h, fluorescence images were captured using confocal microscopy.

### Vector Construction and Rice Transformation

For over-expression the full-length cDNA of Os*FRO1* was cloned into entry vector pENTR-D-TOPO. After sequencing the LR recombination reaction was performed between an attL-containing entry clone and an attR-containing destination vector pEarlyGate 303 to generate the overexpression (Os*FRO1-*Oe*)* vector. For RNA-interference vector, a 300 bp fragment from the Os*FRO1* coding region was amplified and subcloned into entry vector pDONR201 via BR reaction. Then the fragment was inserted into the destination vector pH7GWIWG2(I) in both a sense and anti-sense orientation to get the interference (Os*FRO1*-Ri) vector (Karimi et al., 2002). For expression pattern analysis, the 1800 bp upstream of the ATG start codon of Os*FRO1* was amplified and cloned into pBI101.3 to generate the P_OsFRO1_: GUS vector. All the corresponding primers are listed in Supplementary Table S1.

The Os*FRO1*-Oe, Os*FRO1*-Ri and P_OsFRO1_: GUS vectors were transformed into *Agrobacterium* strain EHA101 or EHA105. Transgenic rice plants for the three constructs were regenerated via *Agrobacterium*-mediated transformation as described (Chen et al., 2003).

### Histochemical β-Glucuronidase (GUS) Staining

T1 transgenic seeds were germinated and grown in nutrient solution with 125 μM Fe (II)-EDTA. Tissues were immersed immediately in staining solution with 1 mM X-Gluc (5-bromo-4-chloro-3-indolyl-b-D-glucuronidase) overnight at 37°C. Afterward, roots were imbedded in 3% (w/v) agarose (Biowest, Spain). Samples were sectioned to a thickness of 30 μm by vibrating microtome (VT 1000 S, Leica, Bensheim, Germany). The Spur’s resin was used to imbed leaves. Sections of 10 μm were cut and the images were observed under a microscope (Eclipse 90i, Nikon, Tokyo, Japan).

### Yeast Fe (III) Chelate Reductase Assay

For assaying Fe (III) reduction activity of OsFRO1 and OsFRO2, the full-length cDNA of OsFRO1, OsFRO2 and AtFRO2 were subcloned into pYES2.0 vector to generate pYES2.0-OsFRO1, pYES2.0-OsFRO2 and pYES2.0-AtFRO2, respectively. Constructs were transformed into *S. cerevisiae* wild-type strain BJ2168 using the lithium acetate method according to the manufacturer’s manual. Yeast transformants were grown following the protocol described previously (Li et al., 2004). Cells were harvested in the mid-log phase, and Fe (III) chelate reductase activity was quantified based on the absorbance at 562 nm (Yi and Guerinot, 1996).

### Total RNA Isolation and Quantitative RT-PCR

Total RNA was extracted from the roots and shoots using TRIzol reagent (Invitrogen, Shanghai, China), according to the manufacturer’s instructions. cDNA was synthesized from 2 μg of total RNA using the PrimeScript^TM^ RT reagent Kit (Takara Bio, Inc., Dalian, China). qRT-PCR were conducted using LightCycler 480 SYBR Green I Master Kit (Roche Diagnostics) on a LightCycler480 machine. The relative expression levels were normalized to the housekeeping gene *OsACTIN1* by the formula 2^−ΔΔCt^. The primers for qRT-PCR analysis are given in Supplementary Table S1.

### Measurement of Element Concentration

To determine the metal concentration in plants, root or shoot samples were dried for 3 days at 80°C. About 0.1 g dry weight was used for digestion. Metal concentration analysis was performed using inductively coupled plasma spectroscopy (ICP-MS, Agilent 7500ce, Santa Clara, CA, United States).

### Histochemical Staining for ROS Production

*In situ* detection of superoxide (O^2−^) in leaves was conducted using NBT staining as described (Höller et al., 2014). In brief, the expanded leaves were excised and immersed in staining solution containing 0.1% (w/v) NBT, 10 mM sodium azide, 50 mM potassium phosphate (pH 6.4). Vacuum infiltrate the leaves for 30 min until they were completely infiltrated. Afterward, incubate the leaves in 10 mL of staining solution (0.1% NBT) for 15 min. ROS formation was visualized by a scanner (CanoScan 9000F MarkII, Tokyo, Japan).

## Results

### Identification of OsFRO1 From the Rice Genome

**FIGURE 1:**
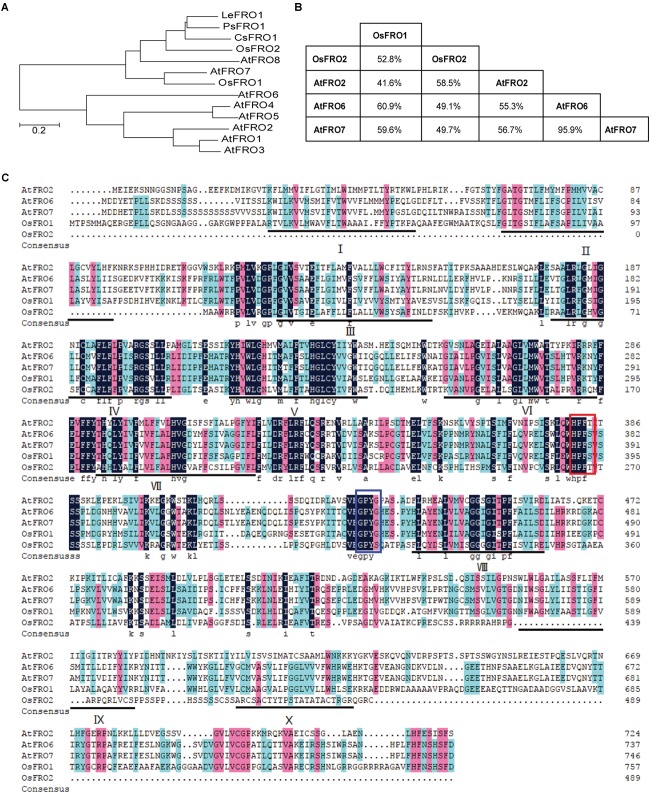
Phylogenetic analysis and alignments of FROs in plants. **(A)** Phylogenetic analysis of FRO proteins in plants. Amino acid sequences were used and the evolutionary history was conducted in MEGA6 via neighbor-joining method. **(B)** The similarity matrix for FRO proteins from rice and Arabidopsis. **(C)** Amino acids sequences alignment of FRO proteins from rice and Arabidopsis. The FAD and NADPH binding site were highlighted by red and blue boxes, respectively. The locations of the 10 potential transmembrane domains were underlined and numbered I-X.

In *Arabidopsis thaliana* there are predicted to be eight *FRO* genes in the genome based on high sequence similarity with *AtFRO2* (Ling et al., 2005). In order to identify *FRO* genes of rice, BLAST searches against the rice genome database using the amino acid sequences of AtFROs were performed. Two putative proteins, named OsFRO1 (LOC_Os04g36720) and OsFRO2 (LOC_Os04g48930) were found as ortyhologs of AtFROs. Phylogenic analysis of FROs in higher plants showed that OsFRO1 displayed higher similarity to AtFRO7, while OsFRO2 clustered together with three characterized FROs, PsFRO1 (Waters et al., 2002), LeFRO1 (Li et al., 2004), CsFRO1 (Waters et al., 2007) in a small group (Figure 1A). OsFRO1 and OsFRO2 have the highest similarity with AtFRO6 (60.9%) and AtFRO2 (58.5%) among the Arabidopsis FROs, respectively (Figure 1B).

Plant FROs were identified based on its sequence similarity to human phagocytic NADPH oxidase gp91phox and yeast ferric-chelate reductases such as FREs (Dancis et al., 1990; Babior, 1995). Similar to FREs and gp91phox, AtFRO2 contains the two conserved motifs HPFT and GPYG that are associated with FAD and NADPH binding sites of the cofactor (Robinson et al., 1999). Amino acid sequence alignment of OsFROs with the AtFROs revealed that both OsFRO1 and OsFRO2 contained the two conservative motifs. The FAD binding site of OsFRO1 shared 100% identity with AtFRO6/7 (HPFS), whereas the FAD motif of OsFRO2 is “HPFT,” identical to AtFRO2. The NADPH binding site of OsFRO1 is GPYS, identical to the other FROs, whereas that of OsFRO2 is GPYT, different from the functional Arabidopsis FROs analyzed (Figure 1C).

The THTMM program was used to predict the membrane topology of two OsFRO proteins, resulting in predictions of 10 transmembrane domains in OsFRO1 and 6 transmembrane domains in OsFRO2 (Supplementary Figure S1). OsFRO2 lacks complete transmembrane domains in its N-terminal and might not be functional. Thus, the study is focused on OsFRO1 for further investigation.

### OsFRO1 Is Localized on the Tonoplast Membrane

**FIGURE 2:**
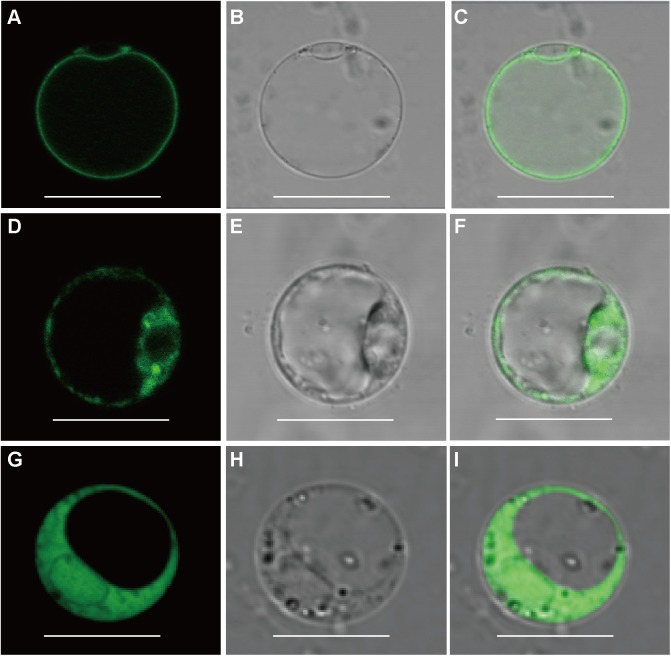
Subcellular localization of OsFRO1 and OsFRO2. **(A)** OsFRO1-GFP expression. **(B)** Bright field image of a protoplast transformed with OsFRO1-GFP. **(C)** Overlay image of **(A,B)**. **(D)** OsFRO2-GFP expression. **(E)** Bright field image of a protoplast transformed with OsFRO2-GFP. **(F)** Overlay image of **(D,E)**. **(G)** 35s-GFP expression. **(H)** Bright field image of a protoplast transformed with 35s-GFP. **(I)** Overlay image of **(G,H)**. Bars = 20 μm.

To determine the subcellular localization of OsFRO1 and OsFRO2 GFP was fused to the C terminus of OsFRO1 and OsFRO2 coding regions. The GFP-fusion constructs were transformed into rice protoplasts isolated from stems and leaves of etiolated seedlings. The green fluorescence signal of OsFRO1 was detected in the vacuolar membrane, whereas that of OsFRO2 located mainly in the cytoplasm (Figure 2). To further confirm the subcellular localization of OsFRO1, a vacuolar membrane marker, OsSPX-MFS3 (Wang et al., 2015), was co-expressed with OsFRO1 in rice protoplasts. The GFP signal mostly overlapped with the mCherry signal of the vacuolar membrane marker (Supplementary Figure S2). Noticeably, the subcellular localization of OsFRO1 was completely different with that of other FROs in plants, since it is the only FRO family member localized to tonoplast to our knowledge. This result indicated that OsFRO1 may play a novel role in Fe homeostasis.

### Expression Profile of *OsFRO1*

**FIGURE 3:**
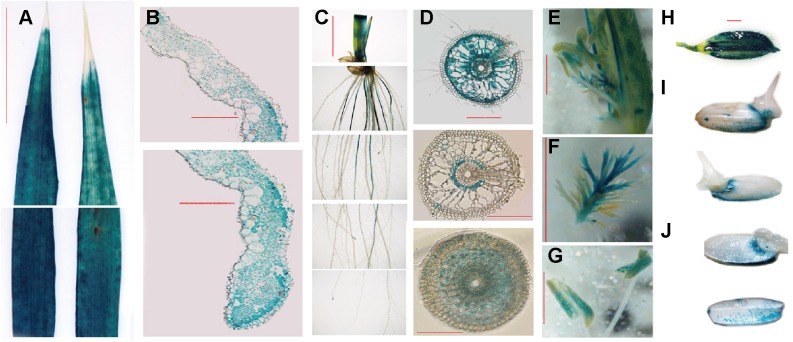
OsFRO1 promoter-driven GUS expression pattern. **(A)** Leaves from 2-week-old seedlings. **(B)** Cross-sections of **(A)**. **(C)** Roots from -week-old seedling. **(D)** Cross-sections of **(C)**. Images of the sections that were close to the root–stem junction (upper panel), 5 cm away from the root tip (middle panel) and 0.5 cm away from the root tip (lower panel). **(E)** GUS staining of the mature flowers. **(F)** Pistil of **(E)**. **(G)** Stamen of **(E)**. **(H)** Seeds before germination. **(I)** Seeds geminated after 1 day. **(J)** Cross-sections of **(I)**. Bar = 1 cm **(A,C)**, Bar = 100 μm **(BD)**, Bar = 1 mm **(E–J)**.

To investigate the spatial expression of *OsFRO1*, transgenic plants carrying the promoter region of *OsFRO1* fused to the *GUS* gene (P_OsFRO1_: GUS) were generated. GUS expression was observed in all tissues, including roots, stems, leaves, flowers and seeds (Figure 3A). Cross-sections of leaves showed that no staining was observed in bulliform cells and part of epidermal cells (Figure 3B). In roots, GUS expression was mainly observed in the mature roots, but not at the young roots. Little staining was detected in root tips (Figure 3C). Cross-sections of roots exhibited that GUS expression was observed throughout root except the epidermal cells (Figure 3D). In the floral organs, GUS was expressed in stigma and pollen (Figure 3E–G). In mature seeds, GUS expression was detected in the glumes (Figure 3H). One day after germination, GUS expression was also detected in the endosperm (Figure 3I,J).

**FIGURE 4:**
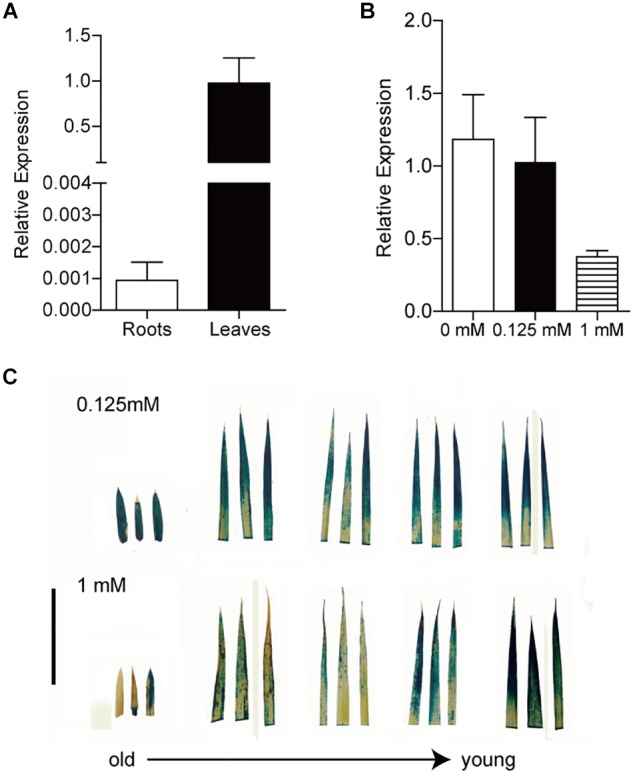
Expression analysis of *OsFRO1* transcript abundance in Rice. **(A)** Transcript abundance of *OsFRO1* in leaves and roots (the expression in leaves was set to 1.0). Seedlings were grown in normal nutrient solution for 3 weeks. Leaves and roots were sampled for RNA extraction. **(B)** Transcript levels of *OsFRO1* under different Fe supply. Two-week-old seedlings cultured in normal nutrient solution (0.125 mM Fe) were transferred to 0 or 1 mM Fe solution for 7 days. Leaves were sampled for RNA extraction. **(C)** GUS staining of P_OsFRO1_: GUS transgenic seedlings under 1 mM Fe treatment. Bar = 3 cm. Data are shown as the mean ± SD (*n* = 3).

To gain further insight into the expression pattern of Os*FRO1* the transcript abundance of Os*FRO1* was analyzed in leaves and roots. Results showed that the basal level of Os*FRO1* in leaves was 1000 times as high as that in roots (Figure 4A). Similar spatial expression pattern can be found from the microarray data retrieved from GENEVESTIGATOR (Supplementary Figure S3). While the mRNA abundance of Os*FRO1* was not affected by Fe deficiency, it is greatly inhibited when plants exposed to solution culture containing excess Fe (Figure 4B). In addition, GUS staining of the P_FRO1_: GUS transgenic plants showed that excess Fe supply significantly suppressed the GUS activity in old leaves, but not in the young leaves (Figure 4C).

### Knock Down of *OsFRO1* Resulted in More Tolerance to Fe Toxicity in Rice Plants

To determine the biological function of OsFRO1 in Fe homeostasis, *OsFRO1* overexpression (Oe) or the RNA interference (Ri) transgenic lines were generated and the transcript abundance of Os*FRO1* in the Oe and Ri plants was determined by quantitative real-time PCR (qRT-PCR) analyses. The *OsFRO1* transcript abundance were 10–40 times higher in the Oe plants, or 2–5 times lower in the Ri plants, compared to that in the wild type (WT) plants (Supplementary Figure S4). Two independent transgenic lines of each construct were selected for further experimental analysis, i.e., Oe-1, Oe-3, Ri-1, and Ri-3.

**FIGURE 5:**
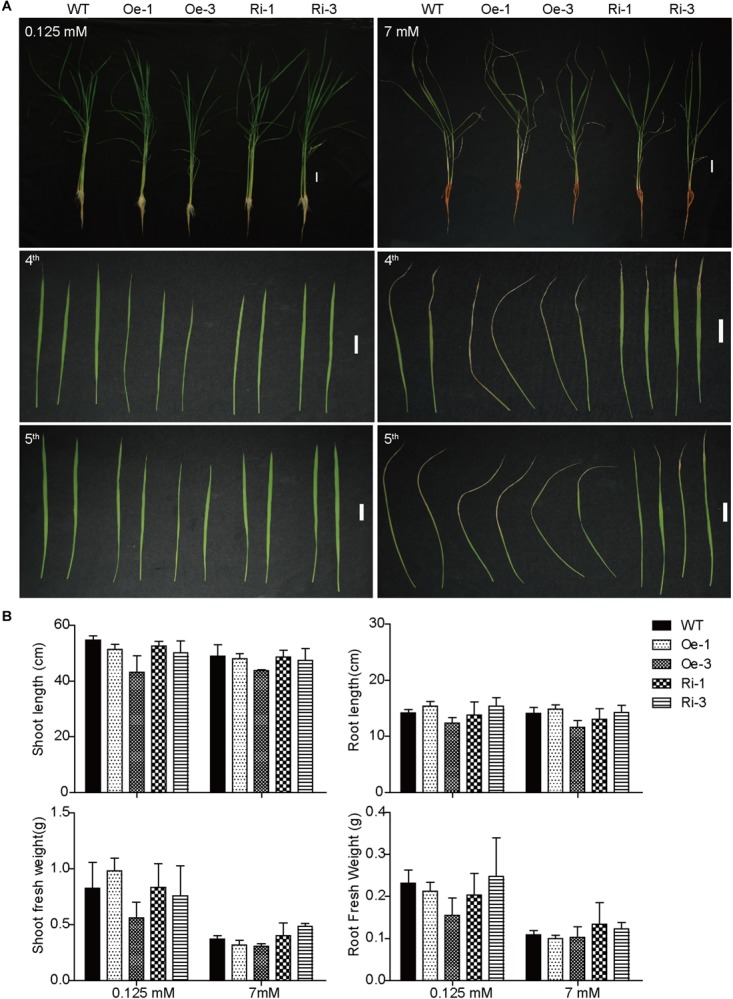
Phenotype analysis of WT and transgenic plants under treatment of excess Fe. **(A)** Growth performance of WT and transgenic plants under normal and Fe excess conditions. Two-week-old plants were grown on nutrient solution supplied with 0.125 or 7 mM Fe (II)-EDTA for 3 days. The fourth and fifth leaves were detached to display their phenotypes. **(B)** Plant length and biomass of WT and transgenic plants under normal and HFe conditions. Bar = 2 cm. Data are shown as the mean and standard deviation (*n* = 3).

To evaluate the effect of OsFRO1 on Fe homeostasis, the transgenic plants were grown under Fe deficiency or Fe excess conditions. Under normal or Fe deficient conditions, there was no significant difference in growth performance and Fe concentrations between the transgenic plants and WT control (Supplementary Figure S5). When treated with excess Fe for 3 days, all the plants showed bronzing in the leaves, and Fe plaques in roots, which are common symptoms of Fe overaccumulation. The 4^th^ and 5^th^ leaves of the WT and transgenic plants were detached and compared. As shown in Figure 5A, *OsFRO1*-Ri lines displayed less bronzing symptom in the leaves compared to WT and Oe plants. No significant differences in plant length or fresh weight were observed between the transgenic plants and WT control (Figure 5B).

**FIGURE 6:**
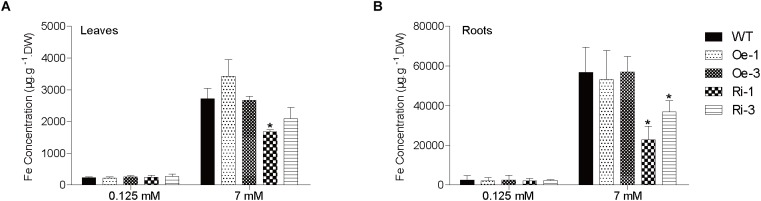
Analysis of Fe concentrations in WT and transgenic plants. Two-week-old plants were grown on nutrient solution supplied with 0.125 or 7 mM Fe (II)-EDTA for 3 days. Fe concentrations in leaves **(A)** and roots **(B)** were measured by ICP-OES. Data are shown as the mean and standard deviation (*n* = 3). Significance of differences compared to WT is indicated by asterisks (Tukey’s ANOVA test; ^∗^*p* < 0.05).

No significant difference was found in leaf and root Fe concentrations among the WT, Ri, and Oe plants when grown in normal Fe supply condition (Figure 6A). Under excess Fe supply condition, the leaf Fe concentrations of the Ri-1 and Ri-3 plants were significantly (*p* < 0.05) or marginally (*p* < 0.1) lower than WT (Figure 6A). In addition, the root Fe concentrations of the Ri-1 and Ri-3 plants were significantly lower than that of WT (Figure 6B). There was no significant difference in the Zn, Mn, Cu concentrations among the leaves and roots of *OsFRO1*-Oe and Ri lines compared to WT (Supplementary Figure S6).

### Knock Down of *OsFRO1* Reduced ROS Accumulation Caused by Excess Fe Stress

**FIGURE 7:**
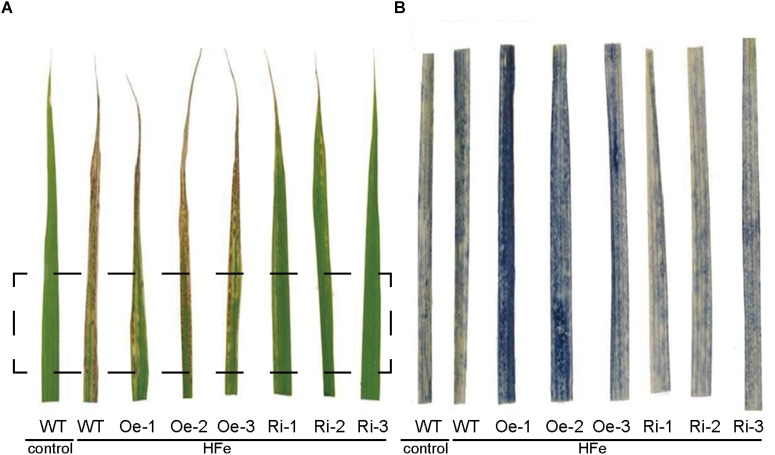
NBT staining of superoxide (O^2−^) in leaves of WT and transgenic rice plants. Three-week-old seedlings cultured in normal nutrient solution were transferred to nutrient solution containing 1 mM Fe and grown for 7 days. Leaves were detached for visualization. **(A)** The leaf-bronzing symptom of WT and transgenic plants under Fe excess conditions. **(B)** The zoom-in region that correspond to rectangle in **(A)** after NBT staining.

Excess Fe resulted an oxidative stress in plants through the formation of ROS by the Fenton reaction (Becana et al., 1998). In order to evaluate the Fe-induced oxidative stress, superoxide (⋅O^2−^) contents representing the major ROS was estimated using NBT staining. Under Fe excess conditions, all the plants showed typical symptoms of Fe overaccumulation (Figure 7A). While the excess Fe supply resulted in significant accumulation of ROS in leaves of WT, Oe and Ri plants, the NBT staining in the *OsFRO1*-Oe leaves was at the highest level (Figure 7B). As contrast, the blue staining in Ri leaves were significantly lower than that in WT and Oe leaves. This NBT staining result is in accordance with the bronzing phenotypes described (Figure 5A).

## Discussion

A rice vacuolar membrane ferric-chelate reductase, OsFRO1, which was down-regulated in shoots under Fe excess conditions was identified in this study. Suppression of the expression of *OsFRO1* by RNAi resulted in tolerant to Fe excess stress in transgenic plants, suggesting that OsFRO1 might be involved in maintaining the Fe balance between the cytoplasm and vacuoles. We propose that OsFRO1 is involved in reducing ferric Fe into ferrous Fe in vacuole, and makes the vacuolar stored Fe available to cytoplasm through Fe (II) or chelated Fe (II) transporters. Under Fe excess condition, the decrease in transcript abundance of Os*FRO1* in the RNAi plants reduced the amount of Fe (II) available for cytoplasm, and therefore alleviates Fe excess toxicity.

Previously, rice FROs were predicted to be non-functional because OsFRO1 and OsFRO2 do not contain perfect conserved HPFT or GPYG motifs compared to Arabidopsis FRO2, which are essential for the binding to the FAD and NADPH in cofactors, respectively (Ishimaru et al., 2006). In the study, we performed a multiple sequence alignment of OsFRO1, OsFRO2, AtFRO2, AtFRO6, and AtFRO7. Results showed that both HPFT and GPYG motifs of OsFRO1 were identical to that of AtFRO6/7 (Figure 1), while the GPYG motif of OsFRO2 is not conserved with the four analyzed Arabidopsis FROs. AtFRO6 was demonstrated to be a functional ferric reductase, which could facilitate the reduction of Fe in tobacco leaves (Li et al., 2011). AtFRO7 was reported to be a chloroplast ferric chelate reductase (Jeong et al., 2008). In this regard, OsFRO1 is likely a functional ferric reductase. To test the reductase activity of OsFRO1 and OsFRO2, we performed a Fe (III)-chelate reductase assay in yeast. Unfortunately, we were not able to detect significant Fe (III)-chelate reductase activity in OsFRO1-expressing or OsFRO2-expressing cells, while AtFRO2-expressing cells showed up to fivefold more Fe (III)-chelate reductase activity than cells transformed with an empty vector (Supplementary Figure S7). The failure of detection of Fe (III)-chelate reductase activity could be caused by the non-plasma membrane localization of OsFRO1 and OsFRO2 in the yeast (data not shown). Because Fe (III)-chelate reductase activity assay requires the membrane impermeant property of bathophenanthroline disulfonic acid, it is difficult to measure the Fe (III) chelate reductase activity of a non-plasma membrane localized FRO. Similar observation was reported in AtFRO7 (Jeong et al., 2008). AtFRO7 is localized in chloroplasts, whose Fe (III) chelate reductase activity could not be detected in the yeast. Further ferric Fe reductase activity assay in yeast may first fuse the OsFRO1 to a protein targeting signal, for instance, the COOH-terminal domain of Ist2p (Jüschke et al., 2005), to facilitate the trafficking of OsFRO1 protein to the plasma membrane in yeast.

The members of FRO family has been well-documented and characterized in fungi, plants, and mammals. Many of them, such as AtFRO2, AtFRO6 in Arabidopsis and FRE1 in yeast, localize to the plasma membrane (Jeong and Connolly, 2009). There are also a subset of FRO family members localizes to internal membranes, such as Arabidopsis AtFRO7 in chloroplast, AtFRO3 and AtFRO8 in mitochondria, and yeast FRE6 in vacuolar membrane, suggesting functional roles in several subcellular compartments (Singh et al., 2007; Jeong et al., 2008; Jain et al., 2014). Vacuoles are considered as the main compartment for excess Fe storage in rice and *Arabidopsis* (Roschzttardtz et al., 2009). Since all known vacuolar transporters, including AtVIT1, AtNRAMP3 and AtNRAMP4, transport Fe across vacuolar membranes in the form of Fe(II) (Lanquar et al., 2005; Kim et al., 2006), it was postulated that Fe reduction might be involved in the vacuolar membrane (Jeong and Connolly, 2009). In yeast, reduction of Fe (III) in vacuoles is carried out by FRE6, which is critical for remobilization of Fe from vacuoles into the cytosol (Raguzzi et al., 1988; Singh et al., 2007). While none of the eight Arabidopsis FROs was reported or predicted to reside on the vacuolar membrane, OsFRO1 was located in the vacuolar membrane in this study. OsFRO1 is likely to reduce Fe (III) into Fe (II) in the vacuolar membrane similar to FRE6 in yeast. The reduced Fe (II) can be transported across the vacuolar membrane into the cytosol by other Fe (II) transporters. Knock down of *OsFRO1* resulted in more tolerance to Fe toxicity in rice plants. The similar phenotype was observed in the rice mutant *MuFRO1* that had a mutation in the *OsFRO1* gene, isolated from a fast neutron mutant library (Ruengphayak et al., 2015). In the Os*FRO1* mutant or knockdown plants, the vacuolar ferric reductase activities are reduced, which might lead to the reduction of the amount of Fe (II) being transported into the cytosol. When exposed to excess Fe, the mutant plants should have less Fe accumulation in the cytosol, and thus showed enhanced tolerance to the excess Fe. The NBT staining showed a significant reduction of ROS accumulation in the *OsFRO1*-Ri leaves, supports this working model for OsFRO1 (Figure 5A).

Os*FRO1*-Ri and Os*FRO1* mutant had a similar decrease in root and shoot Fe concentrations (Figure 6; Ruengphayak et al., 2015). qRT-PCR was carried out to determine the transcript abundance of genes involving in Fe acquisition and translocation, including *IRT1, FRDL1*, *YSL15*, and no significant difference between WT and Os*FRO1-*Ri plants was detected. This indicates the mechanism by which the Fe concentrations were reduced in the *OsFRO1*-Ri or mutant plants merits more investigation to give insight into Fe sensing and response in rice.

In summary, rice FRO, OsFRO1 was shown to function in the vacuolar membrane to converting Fe (III) into Fe (II). Knockdown of Os*FRO1* could alleviate the Fe excess toxicity.

## Data Availability

All datasets generated for this study are included in the manuscript and/or the Supplementary Files.

## Author Contributions

LL carried out most experiments and drafted the manuscript. QK performed the phenotypic analyses and helped in results interpretation. LY helped to conceive the study and its design, and participated in the critical review of the manuscript. HS conceived the study, its design and coordination and helped to draft the manuscript. All authors read and approved the final manuscript.

## Conflict of Interest Statement

The authors declare that the research was conducted in the absence of any commercial or financial relationships that could be construed as a potential conflict of interest.

## Supplementary Material

FIGURE S1 The predicted membrane topology of FROs in Rice and Arabidopsis.

FIGURE S2 Subcellular localization of OsFRO1.

FIGURE S3 Expression levels of OsFRO1 in different tissues **(A)** and different development stages **(B)** based on microarray data retrieved from GENEVESTIGATOR (https://genevestigator.com/gv/).

FIGURE S4 Identification of *OsFRO1* overexpression and RNAi lines.

FIGURE S5 Growth performance of WT and transgenic plants.

FIGURE S6 Analysis of Cu, Zn, Mn concentrations in WT and transgenic plants.

FIGURE S7 Fe (III) chelate reductase assay in yeast cells.

TABLE S1 Primers used in this work.
