# Supplementary material for: A Vacuolar Membrane Ferric-Chelate Reductase, OsFRO1, Alleviates Fe Toxicity in Rice (Oryza sativa L.)
Source: Front Plant Sci. 2019 Jun 4;10:700. doi: 10.3389/fpls.2019.00700 (PMC6558154; doi:10.3389/fpls.2019.00700)
Supplement: FIGURE S1 — The predicted membrane topology of FROs in Rice and Arabidopsis. [file Presentation_1.pptx]

## Slide 1
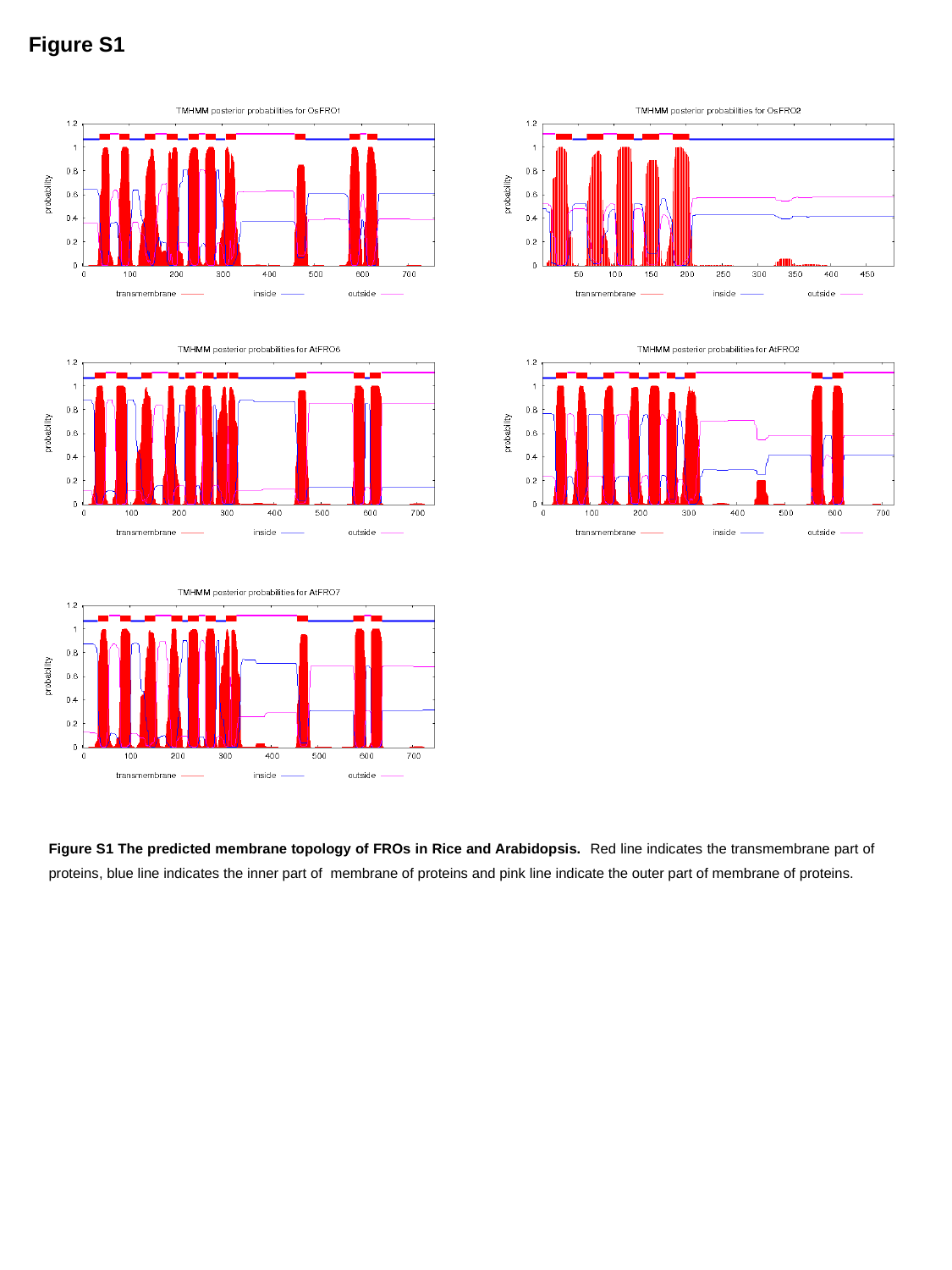

Figure S1
Figure S1 The predicted membrane topology of FROs in Rice and Arabidopsis. Red line indicates the transmembrane part of proteins, blue line indicates the inner part of membrane of proteins and pink line indicate the outer part of membrane of proteins.

## Slide 2
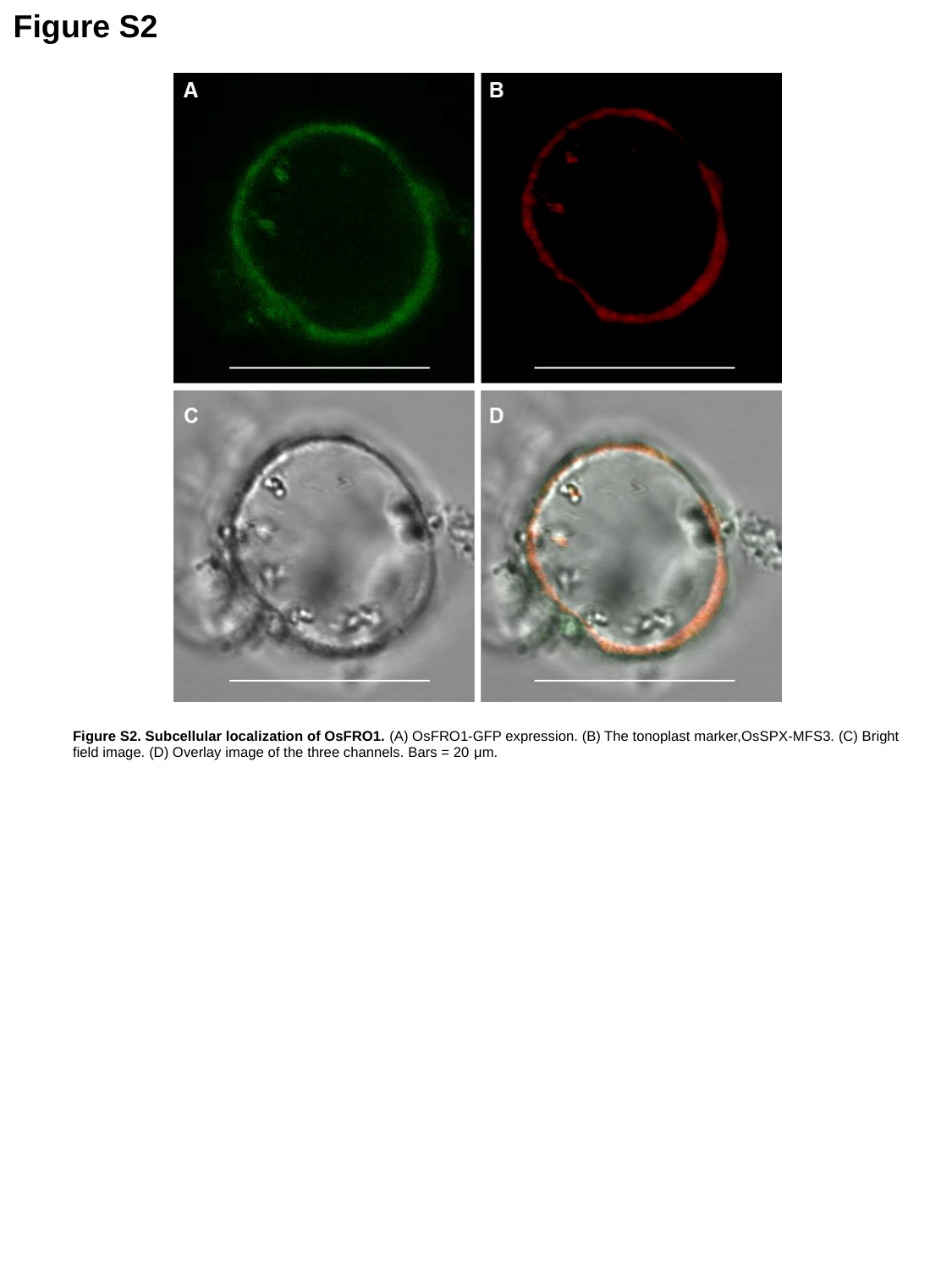

Figure S2
Figure S2. Subcellular localization of OsFRO1. (A) OsFRO1-GFP expression. (B) The tonoplast marker,OsSPX-MFS3. (C) Bright ﬁeld image. (D) Overlay image of the three channels. Bars = 20 μm.

## Slide 3
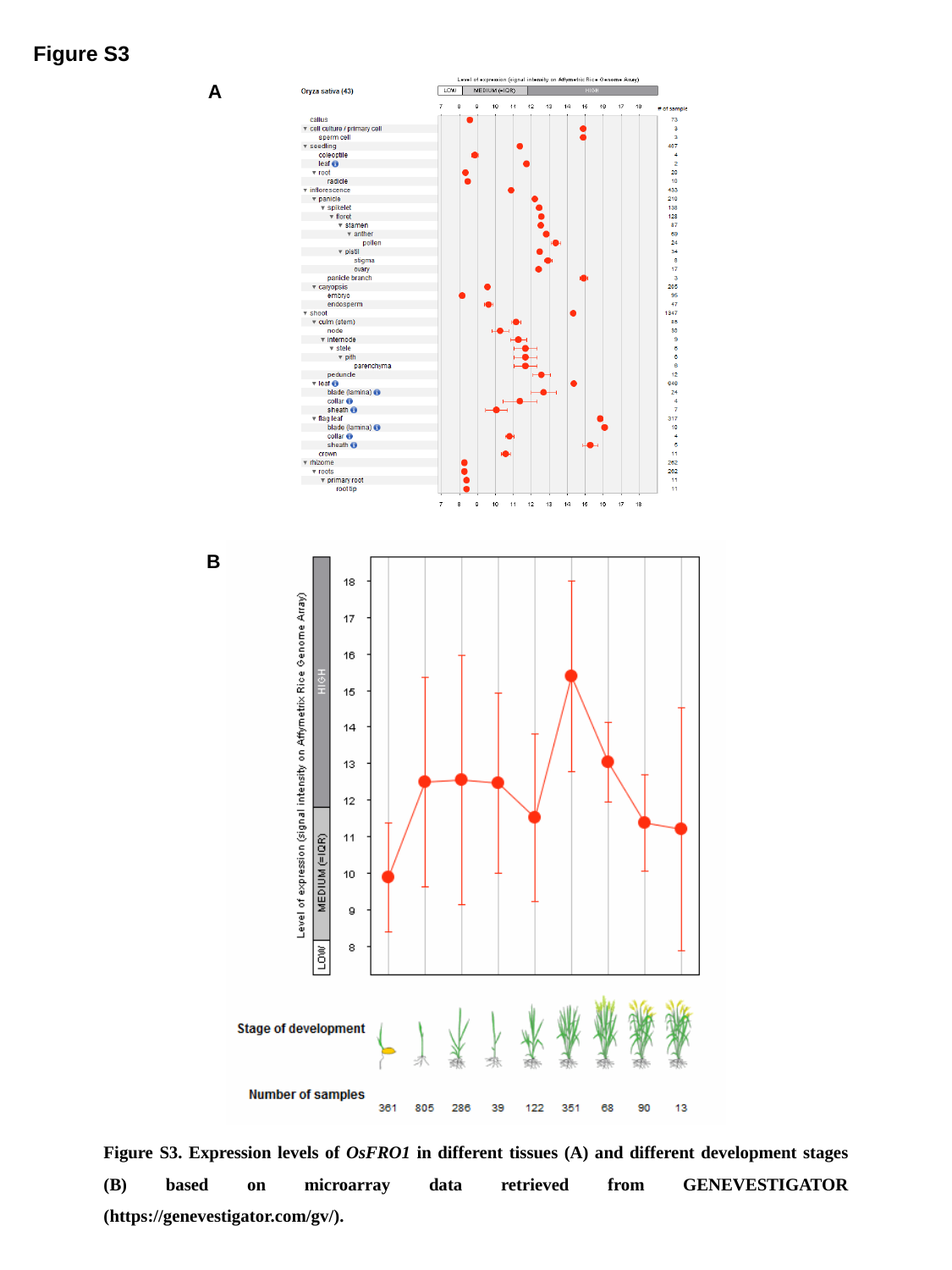

Figure S3
A
B
Figure S3. Expression levels of OsFRO1 in different tissues (A) and different development stages (B) based on microarray data retrieved from GENEVESTIGATOR (https://genevestigator.com/gv/).

## Slide 4
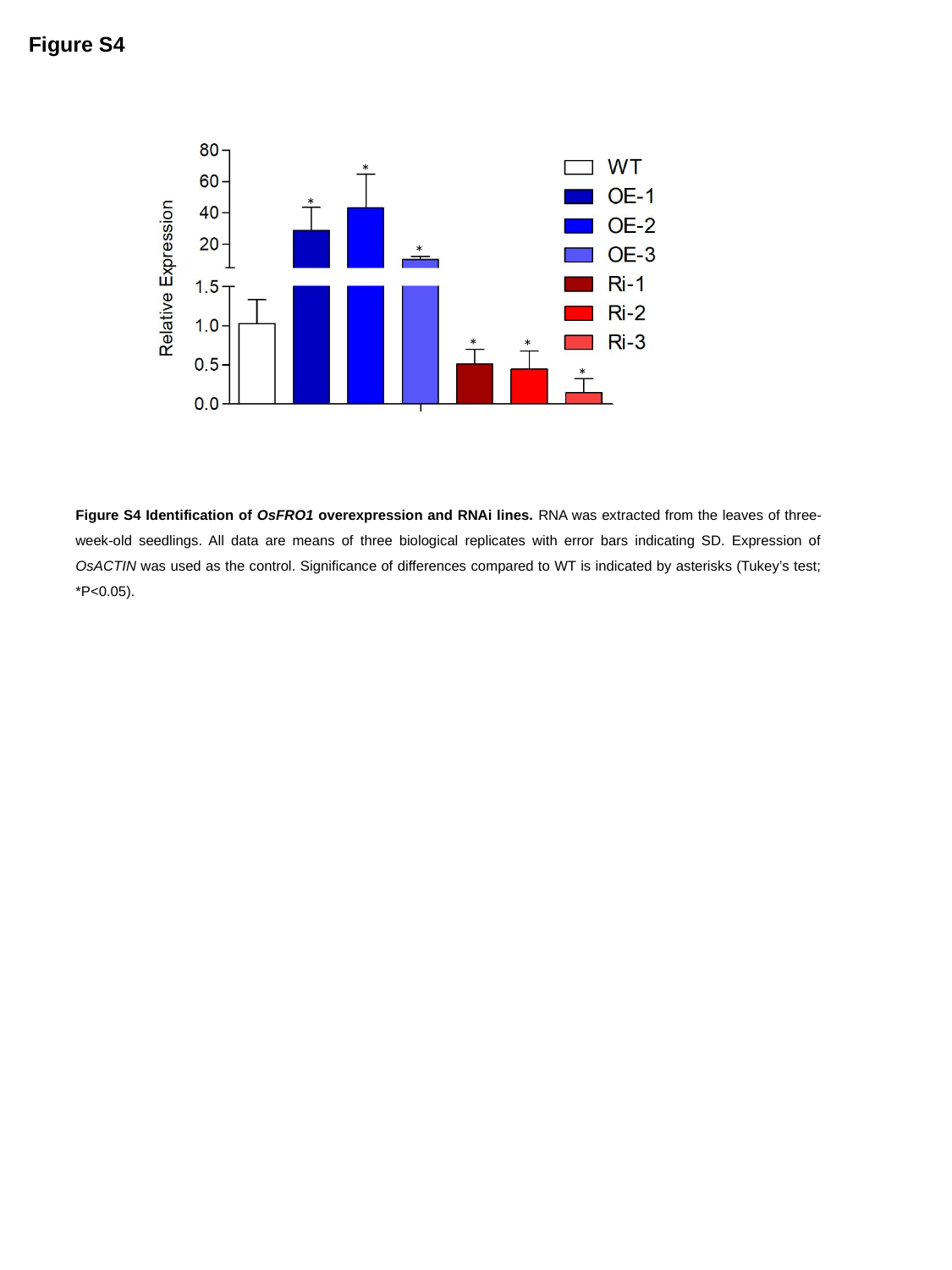

Figure S4
*
*
*
*
*
*
Figure S4 Identification of OsFRO1 overexpression and RNAi lines. RNA was extracted from the leaves of three-week-old seedlings. All data are means of three biological replicates with error bars indicating SD. Expression of OsACTIN was used as the control. Signiﬁcance of differences compared to WT is indicated by asterisks (Tukey’s test; *P<0.05).

## Slide 5
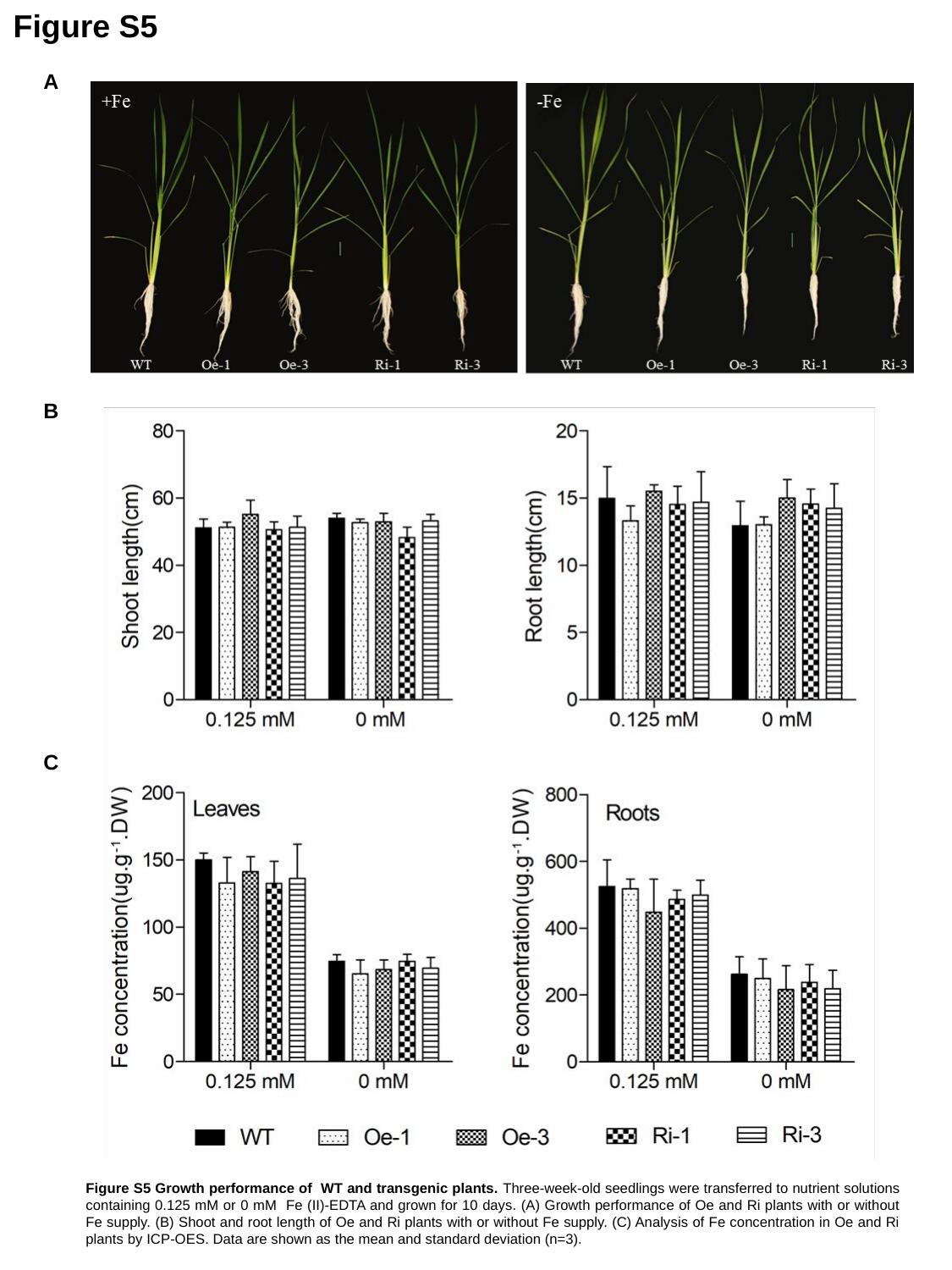

Figure S5
A
B
C
Figure S5 Growth performance of WT and transgenic plants. Three-week-old seedlings were transferred to nutrient solutions containing 0.125 mM or 0 mM Fe (II)-EDTA and grown for 10 days. (A) Growth performance of Oe and Ri plants with or without Fe supply. (B) Shoot and root length of Oe and Ri plants with or without Fe supply. (C) Analysis of Fe concentration in Oe and Ri plants by ICP-OES. Data are shown as the mean and standard deviation (n=3).

## Slide 6
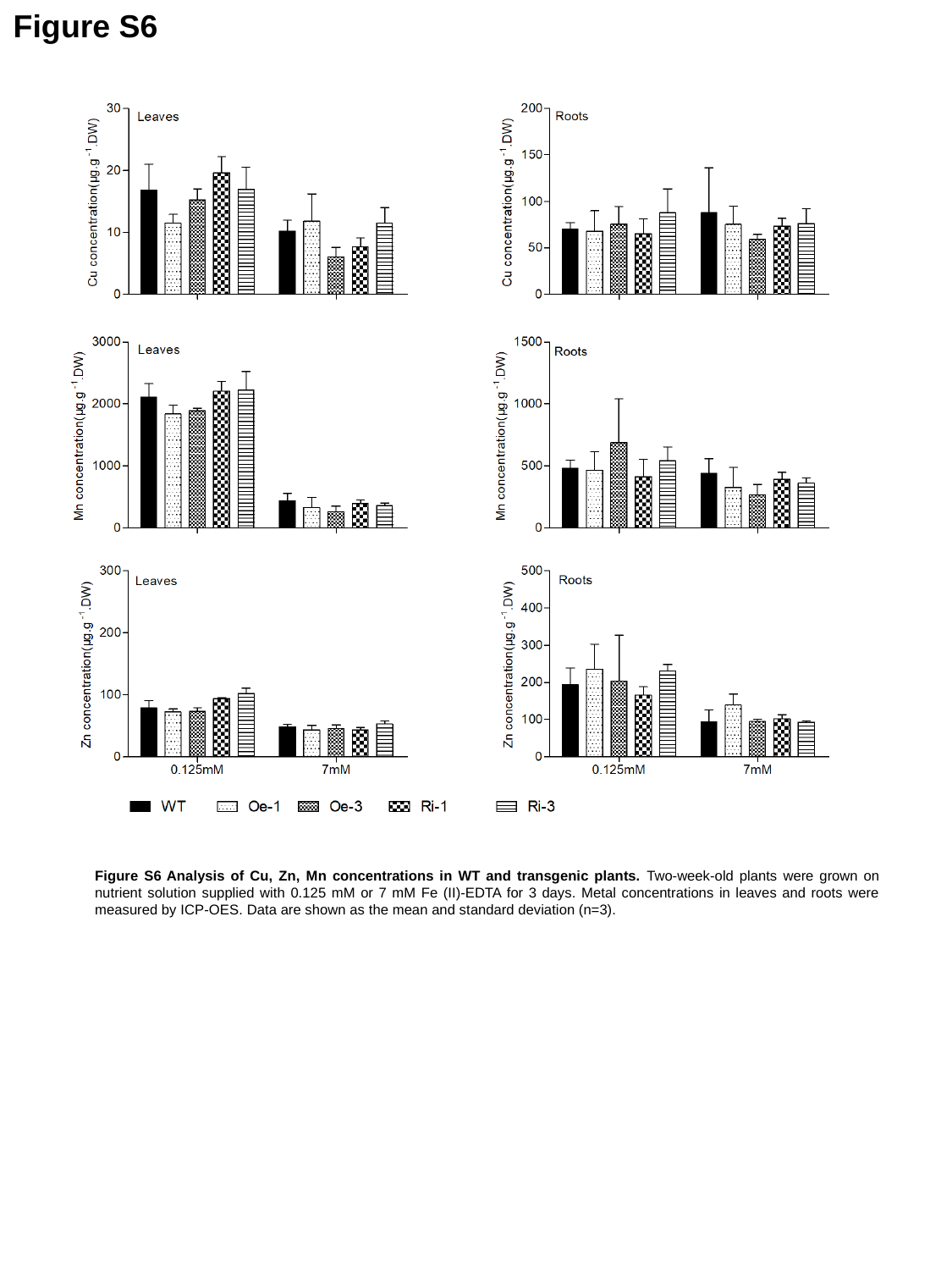

Figure S6
Figure S6 Analysis of Cu, Zn, Mn concentrations in WT and transgenic plants. Two-week-old plants were grown on nutrient solution supplied with 0.125 mM or 7 mM Fe (II)-EDTA for 3 days. Metal concentrations in leaves and roots were measured by ICP-OES. Data are shown as the mean and standard deviation (n=3).

## Slide 7
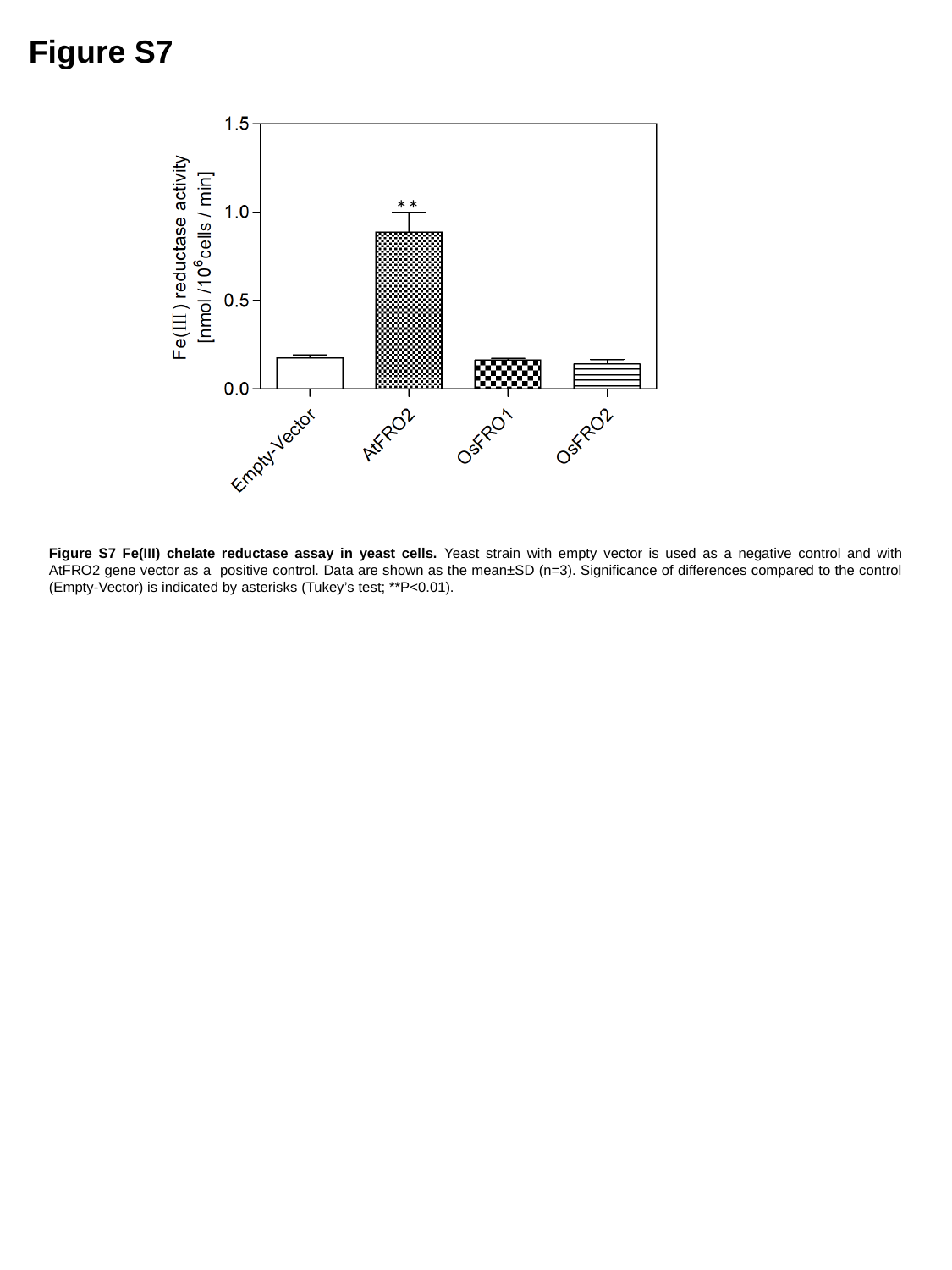

Figure S7
**
Figure S7 Fe(III) chelate reductase assay in yeast cells. Yeast strain with empty vector is used as a negative control and with AtFRO2 gene vector as a positive control. Data are shown as the mean±SD (n=3). Signiﬁcance of differences compared to the control (Empty-Vector) is indicated by asterisks (Tukey’s test; **P<0.01).

## Slide 8
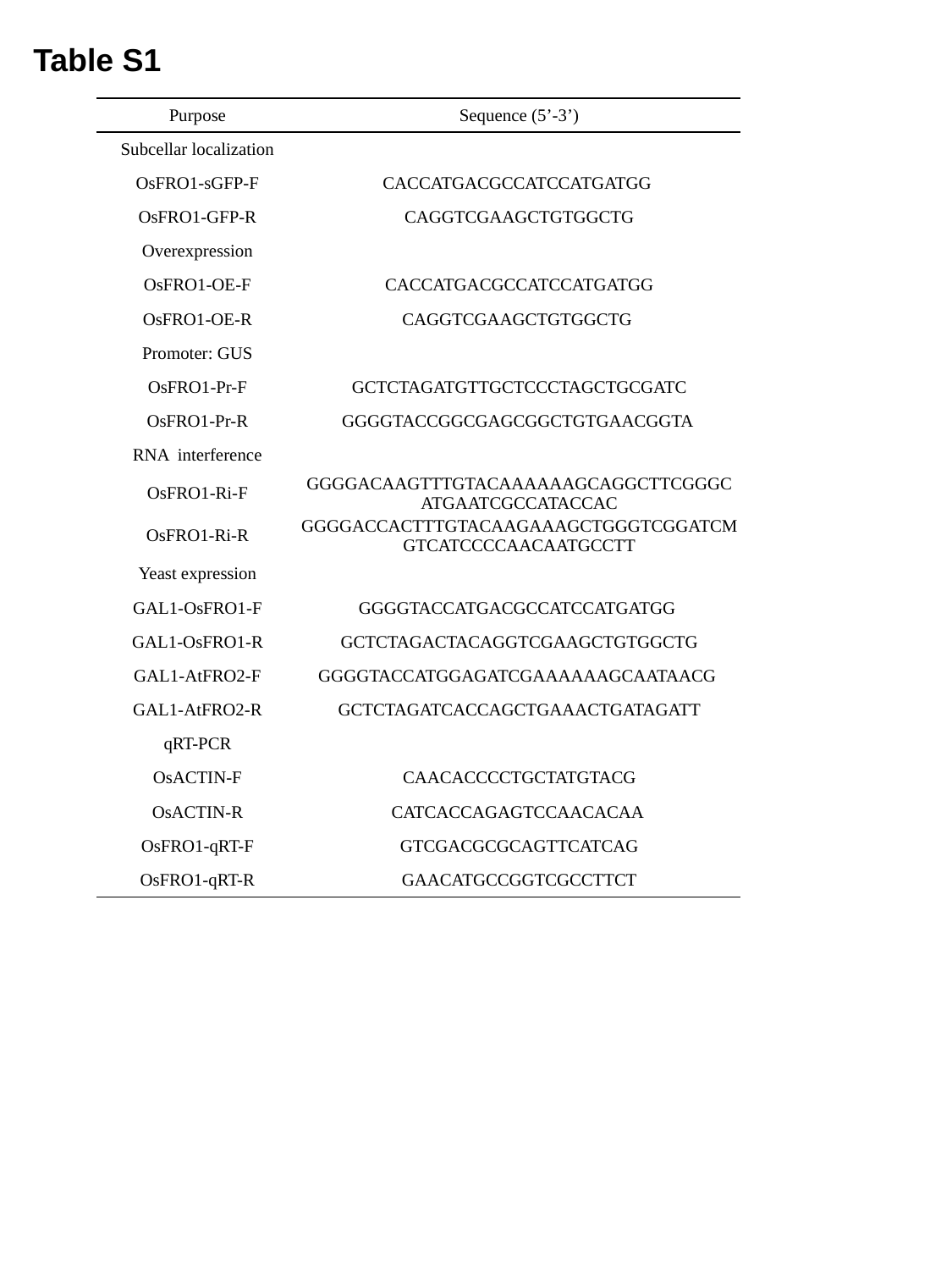

Table S1
| Purpose | Sequence (5’-3’) |
| --- | --- |
| Subcellar localization | |
| OsFRO1-sGFP-F | CACCATGACGCCATCCATGATGG |
| OsFRO1-GFP-R | CAGGTCGAAGCTGTGGCTG |
| Overexpression | |
| OsFRO1-OE-F | CACCATGACGCCATCCATGATGG |
| OsFRO1-OE-R | CAGGTCGAAGCTGTGGCTG |
| Promoter: GUS | |
| OsFRO1-Pr-F | GCTCTAGATGTTGCTCCCTAGCTGCGATC |
| OsFRO1-Pr-R | GGGGTACCGGCGAGCGGCTGTGAACGGTA |
| RNA interference | |
| OsFRO1-Ri-F | GGGGACAAGTTTGTACAAAAAAGCAGGCTTCGGGC ATGAATCGCCATACCAC |
| OsFRO1-Ri-R | GGGGACCACTTTGTACAAGAAAGCTGGGTCGGATCMGTCATCCCCAACAATGCCTT |
| Yeast expression | |
| GAL1-OsFRO1-F | GGGGTACCATGACGCCATCCATGATGG |
| GAL1-OsFRO1-R | GCTCTAGACTACAGGTCGAAGCTGTGGCTG |
| GAL1-AtFRO2-F | GGGGTACCATGGAGATCGAAAAAAGCAATAACG |
| GAL1-AtFRO2-R | GCTCTAGATCACCAGCTGAAACTGATAGATT |
| qRT-PCR | |
| OsACTIN-F | CAACACCCCTGCTATGTACG |
| OsACTIN-R | CATCACCAGAGTCCAACACAA |
| OsFRO1-qRT-F | GTCGACGCGCAGTTCATCAG |
| OsFRO1-qRT-R | GAACATGCCGGTCGCCTTCT |
